# Association of mono-2-ethylhexyl phthalate with adverse outcomes in chronic hemodialysis patients

**DOI:** 10.1007/s11356-023-30814-z

**Published:** 2023-11-08

**Authors:** Chia-Lin Wu, Yu-Wei Fang, Yi-Chou Hou, Kuo-Cheng Lu, Wen-Hsin Tsai, Ping-Hsun Lu, Tzong-Shyuan Lee, Ko-Lin Kuo

**Affiliations:** 1 Department of Post-Baccalaureate Medicine, College of Medicine, National Chung Hsing University, Taichung, 40227 Taiwan; 2 School of Medicine, Chung Shan Medical University, Taichung, 40201 Taiwan; 3 Division of Nephrology, Department of Internal Medicine, Changhua Christian Hospital, Changhua, 50006 Taiwan; 4 Division of Nephrology, Department of Internal Medicine, Shin-Kong Wu Ho-Su Memorial Hospital, Taipei, 111045 Taiwan; 5 School of Medicine, Fu Jen Catholic University, New Taipei City, 242062 Taiwan; 6 Division of Nephrology, Department of Medicine, Cardinal-Tien Hospital, New Taipei City, 23148 Taiwan; 7 Division of Nephrology, Department of Medicine, Fu Jen Catholic University Hospital, New Taipei City, 24352 Taiwan; 8 Division of Nephrology, Department of Medicine, Taipei Tzu Chi Hospital, Buddhist Tzu Chi Medical Foundation, New Taipei City, 23142 Taiwan; 9 School of Medicine, Buddhist Tzu Chi University, Hualien, 97004 Taiwan; 10 Department of Pediatrics, Taipei Tzu Chi Hospital, Buddhist Tzu Chi Medical Foundation, New Taipei City, 23142 Taiwan; 11 Department of Chinese Medicine, Taipei Tzu Chi Hospital, Buddhist Tzu Chi Medical Foundation, New Taipei City, 23142 Taiwan; 12 School of Post-Baccalaureate Chinese Medicine, Tzu Chi University, Hualien, 97004 Taiwan; 13 Graduate Institute and Department of Physiology, College of Medicine, National Taiwan University, Taipei, 106319 Taiwan

**Keywords:** Adverse outcome, Di-2-ethylhexyl phthalate, Hemodialysis, Mono-2-ethylhexyl phthalate, Mortality

## Abstract

Phthalate exposure is widespread and has a global impact. Growing evidence shows that mono-2-ethylhexyl phthalate (MEHP) exposure has a negative impact on human health. However, whether MEHP exposure is associated with mortality and other adverse outcomes in hemodialysis patients remains unknown. This study prospectively enrolled 217 patients on maintenance hemodialysis from June 30, 2021, to August 16, 2022. Baseline serum MEHP, di-2-ethylhexyl phthalate (DEHP), and indoxyl sulfate (IS) concentrations were measured. Primary endpoints were all-cause mortality or composite adverse outcomes, including all-cause death plus hospitalization due to cardiovascular disease, heart failure, stroke, infection, or cancer. Serum MEHP concentrations were positively associated with DEHP but not indoxyl sulfate concentrations in hemodialysis patients. Additionally, serum MEHP concentrations were significantly and independently associated with all-cause mortality and composite adverse outcomes (adjusted hazard ratios [HRs], 1.04 and 1.03 per ng/mL, 95% confidence intervals [CIs], 1.01–1.07 and 1.00–1.05; p = 0.016 and 0.015, respectively). We found a cutoff value of MEHP for predicting both endpoints. Patients with serum MEHP concentrations of ≥ 41.8 ng/mL had much higher risks for all-cause mortality and composite adverse outcomes (adjusted HRs, 39.2 and 13; 95% CIs, 2.44–65.7 and 2.74–61.4; p = 0.011 and 0.001, respectively). MEHP exposure is significantly associated with higher risks for all-cause mortality and composite adverse outcomes. Hemodialysis patients with serum MEHP concentrations above 41.8 ng/mL had much poorer prognoses regarding both outcomes.

**Supplementary Information:**

The online version contains supplementary material available at 10.1007/s11356-023-30814-z.

## Introduction

End-stage renal disease (ESRD) is a global health burden that greatly impacts human health. The population of patients with ESRD continues to grow worldwide (McCullough et al. 2019; Thurlow et al. 2021). Patients with uremia still have very high risks for premature death, cardiovascular diseases, and other complications, such as infection, malnutrition, sarcopenia, and malignancy, despite dialysis treatment (Collins et al. 2015). With progress in knowledge about the traditional and nontraditional risk factors for uremia and advances in medications and interventional therapy, the mortality and morbidity rates of dialysis patients have been falling in the past two decades (Collins et al. 2015). However, death and complication rates remain high in dialysis patients and cannot be fully explained by current risk factors and dialysis modalities. Thus, it is necessary to explore currently unknown nontraditional contributing factors for high rates of mortality and adverse clinical outcomes in dialysis patients.

Phthalates are substances that are added to plastics to increase flexibility, elasticity, durability, and transparency and are mainly used as plasticizers (Sree et al. 2023). Exposure to phthalates is widespread in most developed countries. Di-2-ethylhexyl phthalate (DEHP) and mono-2-ethylhexyl phthalate (MEHP) are two major phthalates and have been considered the culprits for endocrine disorders. DEHP has been reported to be linked to diseases such as obesity (Choi et al. 2014), atherosclerosis (Lind and Lind 2011), and high blood pressure in several populations (Trasande et al. 2013). DEHP has also been reported to be the material for hemodialysis-associated plastic devices or instruments (Guo et al. 2020). We recently reported that high DEHP exposure offsets the beneficial effects of statins in dialysis patients and in their endothelial cells (Guo et al. 2020). MEHP is derived from DEHP in the gut and is considered the major active metabolite of DEHP (Koch et al. 2006). A recent population-based longitudinal cohort study revealed that MEHP is significantly associated with an increased risk of cardiovascular mortality (Trasande et al. 2022). However, whether MEHP is also linked to poor prognosis in patients on maintenance hemodialysis remains unknown. Therefore, the current study aimed to explore the association of exposure to MEHP with adverse clinical outcomes in hemodialysis patients.

## Methods and Materials

### Participants

We prospectively recruited 363 participants on chronic hemodialysis at the dialysis unit in the network of Taipei Tzu Chi Hospital or Shin-Kong Wu Ho-Su Memorial Hospital from June 30, 2021, to August 16, 2022. All participants had undergone hemodialysis for at least three months and had no hospitalization within three months. Blood was sampled to assess the baseline concentrations of phthalates (DEHP and MEHP) and indoxyl sulfate (IS) in these patients. We excluded 146 participants whose baseline phthalates and IS concentrations were not determined together (Supplementary Fig. S1). Then, the participants were followed up until the occurrence of primary clinical endpoints or the end of the study. The study was approved by the Institutional Review Board of Taipei Tzu Chi Hospital (approval number: 09-X-129) and Shin-Kong Wu Ho-Su Memorial Hospital (approval number: 20200907R).

### Clinical outcome measures

The primary clinical endpoints were all-cause mortality and composite adverse outcomes. A composite adverse outcome was defined as hospitalization due to cardiovascular disease, heart failure, stroke, infection, or cancer and all-cause death.

### Biochemistry measurements

Blood sampling was performed after a 12-h fasting period. Serum biochemical measurements were performed by using a biochemistry analyzer (Dimension® RXL Max® integrated chemistry system, Siemens, Erlangen, Germany).

### Measurements of DEHP, MEHP, and IS

Serum concentrations of DEHP, MEHP, and IS were determined at the beginning of this study. Blood samples were collected in glass tubes and centrifuged at 900 × g for 10 min at 4 °C immediately after collection. Serum samples were frozen at -80 °C until analysis. Serum concentrations of DEHP and MEHP were determined by liquid chromatography/tandem mass spectrometry (Agilent 1100 HPLC system, CTC PAL Autosampler, and SCIEX 4000 Triple Quadrupole Mass Spectrometer). Human serum indoxyl sulfate (IS) concentrations were determined by using an enzyme-linked immunosorbent assay kit (FineTest, Wuhan, China) according to the manufacturer's instructions.

### Statistical analysis

Data are expressed as the number (percentage), mean (standard deviation, SD), or median (interquartile range) as appropriate. We used the Kolmogorov–Smirnov test to determine the normality of continuous variables. A variable whose distribution rejected the null hypothesis of normal distribution was compared using the nonparametric Kruskal–Wallis test; otherwise, it was compared using a parametric one-way ANOVA test. The proportions among the groups were compared by Chi-squared test. In addition, Fisher’s exact test was used if there was an observed value of less than five for categorical variables. Correlations among DEHP, MEHP, and IS were assessed by Pearson’s correlation coefficient. The Kaplan‒Meier method was used to compare the incidence of all-cause mortality or composite adverse outcomes among the study groups. We used Cox proportional hazard (PH) regression to determine the hazard ratio (HR) of variables of interest with all-cause mortality and composite adverse outcomes. The goodness of fit of the multivariate Cox PH models for all-cause mortality and composite adverse outcomes was determined by using Harrell’s C-index. Harrell’s C-index values were all above 0.8 for both endpoints (Supplementary Fig. S2 and Fig. S3). The cutoff value of MEHP for predicting adverse outcomes in hemodialysis patients was determined by the time-dependent receiver operating characteristic (ROC) curve (the R packages survivalROC and risksetROC). Multivariate-adjusted restricted cubic spline Cox PH regression was used to reveal the relationship between circulating MEHP concentrations and the composite adverse outcome (the R packages rms and splines). A two-tailed p-value of less than 0.05 was considered statistically significant. All statistical analyses were performed by using SAS software (version 9.4, SAS Institute Inc., Cary, NC, USA), R software (version 4.2.1, the R Foundation), and STATA (version 15.1, Stata Corp, College Station, TX, USA).

## Results

### Patient characteristics

A total of 217 patients on chronic hemodialysis were included in this study (Supplementary Fig. S1). The mean age was 66.1 ± 12.2 years, the mean dialysis vintage was 5.9 ± 6.6 years, and the mean serum albumin concentration was 3.9 ± 0.3 g/dL; 52.5% were male; 54.5% had diabetes mellitus; 79.7% had hypertension; 25.8% had cardiovascular diseases; and 92.6% patients had adequate dialysis (Kt/V > 1.2).

### Associations between circulating phthalates and protein-bound uremic toxins

**Fig. 1:**
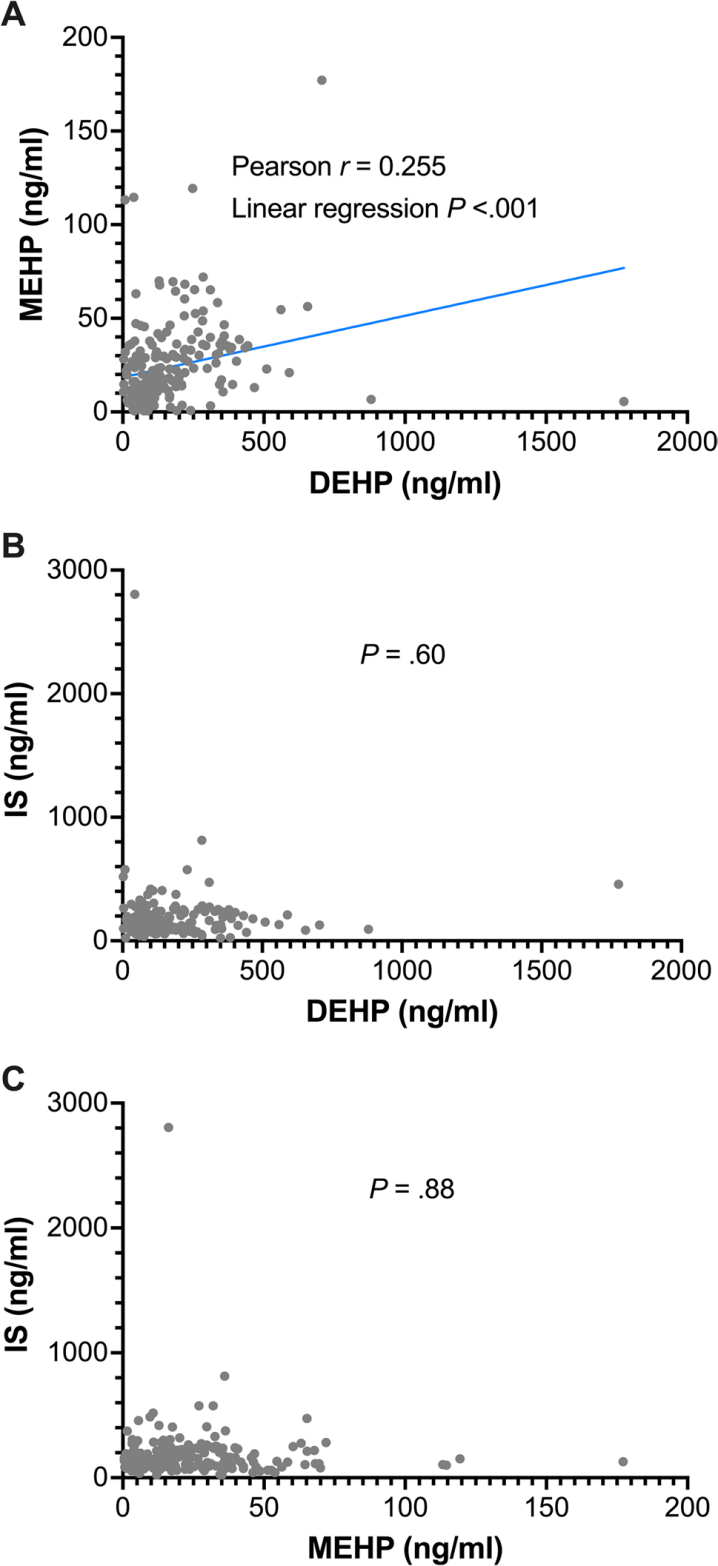
Associations between circulating DEHP, MEHP, and IS concentrations. DEHP, di-2-ethylhexyl phthalate; IS, indoxyl sulfate; MEHP, mono-2-ethylhexyl phthalate.

Serum MEHP concentrations were positively and linearly correlated with DEHP concentrations (Pearson *r* = 0.255, *p* = 0.0002; Fig. 1A). However, circulating concentrations of the major protein-bound uremic toxin, IS, were not significantly correlated with DEHP (*p* = 0.6; Fig. 1B) and MEHP (*p* = 0.88; Fig. 1C) concentrations in hemodialysis patients.

### Exploring the cutoff value of MEHP for predicting adverse clinical outcomes in hemodialysis patients

Time-dependent ROC analysis revealed the optimal cutoff value of 41.8 ng/mL for predicting adverse clinical outcomes in hemodialysis patients (Supplementary Fig. S4). The patients were classified into three groups by circulating MEHP concentration (Supplementary Fig. S1): “low” included patients with MEHP concentrations below the 25^th^ percentile (< 7.89 ng/ml); “modestly elevated” included patients with MEHP concentrations between the 25^th^ percentile and the cutoff value (7.89 to 41.79 ng/ml); and “high” included patients with MEHP concentrations above the cutoff value (≧41.8 ng/ml). Compared with the other MEHP groups, the high MEHP group had similar age, dialysis vintage, and serum albumin concentrations and similar proportions of male sex, diabetes, hypertension, cardiovascular disease, and adequate dialysis (Kt/V > 1.2) but had higher circulating MEHP and DEHP concentrations (Table 1). Additionally, significantly higher proportions of subjects in the high MEHP group developed adverse clinical outcomes during the follow-up period (death and the composite adverse outcome, both *p* = 0.002; Table 1).

**Table 1:** Baseline characteristics and clinical outcomes of the study participants stratified by circulating MEHP levels

	Low MEHP	Modestly elevated MEHP	High MEHP	p-value
	< 7.89 ng/ml (< 25^th^ percentile)	Between 7.89 to 41.79 ng/ml (25^th^ percentile to the cutoff value)	≧41.8 ng/ml (≧cutoff value)	
No. of participants	54	135	28	
Age, year, mean (SD)	65.6 (11.6)	66.1 (12.1)	67 (13.7)	0.64^a^
Age ≧65 years, n (%)	27 (50)	77 (57)	16 (57.1)	0.66^b^
Male, n (%)	30 (55.6)	70 (51.9)	14 (50)	0.86^b^
Diabetes mellitus, n (%)	29 (53.7)	74 (54.8)	15 (53.6)	0.99^b^
Hypertension, n (%)	46 (85.2)	103 (76.3)	24 (85.7)	0.27^b^
CVD, n (%)	17 (31.5)	32 (23.7)	7 (25)	0.54^b^
Dialysis vintage, year, mean (SD)	5.1 (4.6)	6.1 (7)	6.8 (8.1)	0.24^a^
Vintage > 5 years, n (%)	22 (40.7)	43 (31.9)	10 (35.7)	0.51^b^
MEHP, ng/ml, median (IQR)	4 (2.2–5.5)	20.1 (14–29.9)	59.4 (47.9–68.8)	< 0.001^c^
DEHP, ng/ml, median (IQR)	70 (50.5–95)	123 (65.5–220)	219.5 (130–284.8)	0.001^c^
Total IS, ng/ml, median (IQR)	113.4 (85.6–168.9)	164.3 (99.2–235.7)	113.4 (82.3–176.3)	0.005^c^
Kt/v > 1.2, (%)	49 (90.7)	128 (94.8)	24 (85.7)	0.20^a^
Serum albumin, mg/dl, mean (SD)	3.8 (0.4)	3.9 (0.3)	3.9 (0.2)	0.14^a^
Clinical outcomes				
Death, n (%)	1 (1.9)	2 (1.5)	4 (14.3)	0.002^d^
Composite adverse outcome^e^, n (%)	3 (5.6)	5 (3.7)	6 (21.4)	0.002^d^

Abbreviations: CVD, cardiovascular disease; DEHP, di-2-ethylhexyl phthalate; IQR, interquartile range; IS, indoxyl sulfate; MEHP, mono-2-ethylhexyl phthalate; SD, standard deviation

^a^ ANOVA test

^b^ Chi-squared test

^c^ Kruskal–Wallis test

^d^ Fisher’s exact test

^e^ The composite adverse outcome includes hospital admission due to cardiovascular disease, heart failure, stroke, infection, or cancer, and all-cause mortality

### Association of circulating MEHP with survival and composite adverse outcomes

Table 2 shows the association between adverse outcomes and potentially relevant variables in hemodialysis patients. Serum MEHP and albumin were associated with higher (HR, 1.02 per ng/mL; *p* = 0.018) and lower HRs (HR, 0.17 per g/dL; *p* = 0.032) for all-cause mortality in univariate Cox PH analysis, respectively. In multivariate Cox PH analysis, serum MEHP and albumin remained significantly associated with higher (4% increase in HR per ng/mL, *p* = 0.016) and lower HRs (99% decrease in HR per g/dL, *p* = 0.027) for all-cause mortality, respectively. Regarding the composite adverse outcome, serum MEHP (3% increase in HR per ng/mL, *p* = 0.015), age above 65 years (HR, 6.25; *p* = 0.037), and male sex (HR, 4.24; *p* = 0.044) were independently associated with higher HRs in multivariate Cox PH analysis.

**Table 2:** Association of mortality and composite adverse clinical outcome with phthalates and other baseline clinical parameters

	All-cause mortality				Composite adverse outcome^a^			
	Crude HR	p-value	Adjusted HR	p-value	Crude HR	p-value	Adjusted HR	p-value
MEHP (per ng/ml)	1.02 (1.00–1.04)	0.018	1.04 (1.01–1.07)	0.016	1.01 (1.00–1.03)	0.12	1.03 (1.00–1.05)	0.015
DEHP (per ng/ml)	1.00 (0.99–1.00)	0.79	1.00 (0.99–1.00)	0.41	1.00 (0.99–1.00)	0.51	1.00 (0.99–1.00)	0.23
Total IS (per ng/ml)	0.99 (0.98–1.00)	0.11	0.98 (0.97–1.00)	0.10	0.99 (0.99–1.00)	0.14	0.99 (0.99–1.00)	0.19
Age > 65 years	4.96 (0.60–41.2)	0.14	6.26 (0.35- 113)	0.21	5.11 (1.14–22.8)	0.033	6.25 (1.12–34.9)	0.037
Male gender	1.20 (0.27–5.38)	0.81	1.88 (0.29–12.4)	0.51	3.38 (0.94–12.1)	0.06	4.24 (1.04–17.3)	0.044
Diabetes mellitus	2.12 (0.41–11.0)	0.37	3.18 (0.49–20.7)	0.23	2.15 (0.67–6.85)	0.2	1.92 (0.55–6.75)	0.31
Hypertension	—^b^	—^b^	—^b^	—^b^	1.52 (0.34–6.80)	0.58	2.74 (0.33–22.6)	0.35
CVD	1.15 (0.22–5.94)	0.87	0.34 (0.03–3.40)	0.36	2.22 (0.77–6.40)	0.14	1.70 (0.54–5.35)	0.36
Vintage > 5 years	1.45 (0.33–6.49)	0.62	1.22 (0.21–7.06)	0.82	1.94 (0.68–5.54)	0.21	2.39 (0.69–8.32)	0.17
Kt/v > 1.2	0.46 (0.05–3.79)	0.47	3.43 (0.12–96.3)	0.47	1.01 (0.13–7.70)	0.99	1.69 (0.10–27.5)	0.71
Serum albumin, g/dl	0.17 (0.03–0.86)	0.032	0.01 (0.00–0.60)	0.027	0.72 (0.14–3.66)	0.69	1.31 (0.13–13.1)	0.82

Abbreviations: CVD, cardiovascular disease; DEHP, di-2-ethylhexyl phthalate; HR, hazard ratio; IS, indoxyl sulfate; MEHP, mono-2-ethylhexyl phthalate

^a^ The composite adverse outcome includes hospital admission due to cardiovascular disease, heart failure, stroke, infection, or cancer, and all-cause mortality

^b^ Did not converge

**Fig. 2:**
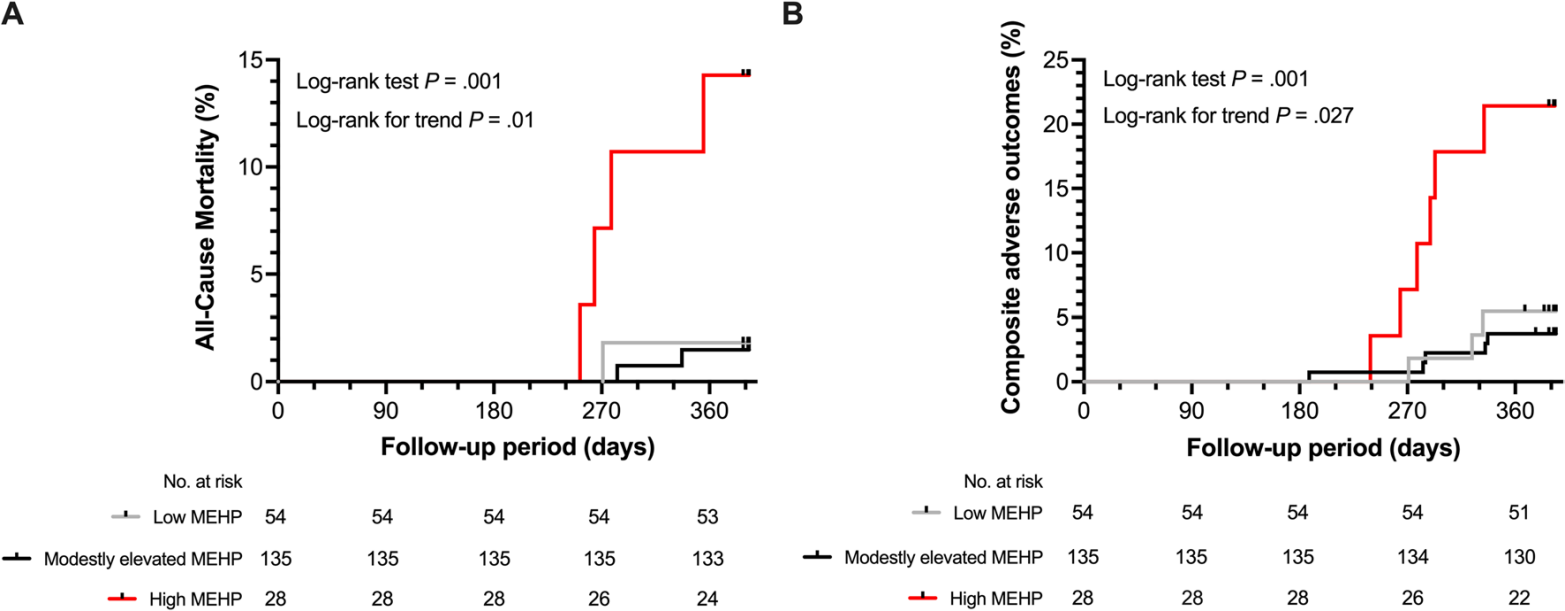
The associations between baseline MEHP groups and (A) all-cause mortality and (B) composite adverse outcomes. MEHP groups were classified by circulating MEHP concentrations: Low, < 7.89 ng/ml; Modestly elevated, between 7.89 to 41.79 ng/ml; High, ≥ 41.8 ng/ml. MEHP, mono-2-ethylhexyl phthalate

**Fig. 3:**
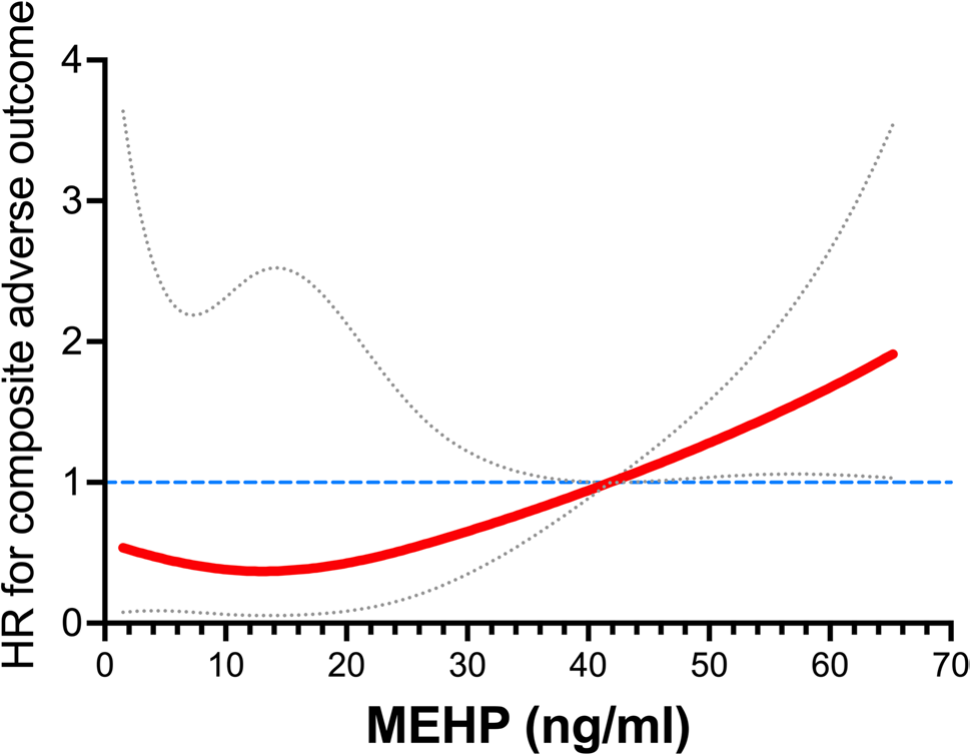
The relationship between the hazard ratios for composite adverse outcomes and circulating MEHP concentrations in the restricted cubic spline regression model. The red solid line indicates adjusted hazard ratios and the 95% confidence intervals are within the space between the gray dotted lines. The hazard ratios have been adjusted to DEHP, IS, age, sex, diabetes, hypertension, cardiovascular disease, dialysis vintage, dialysis adequacy (Kt/V), and serum albumin. DEHP, di-2-ethylhexyl phthalate; HR, hazard ratio; IS, indoxyl sulfate; MEHP, mono-2-ethylhexyl phthalate.

Serum MEHP greater than the cutoff value (41.8 ng/mL) had a large negative impact on survival and the composite adverse outcome. Kaplan‒Meier curves revealed that the high MEHP group had a significantly higher mortality rate than the other two groups (log-rank test *p* = 0.001 and p-value for trend = 0.01; Fig. 2A). In addition, the high MEHP group had a substantially higher risk for all-cause mortality in multivariate Cox PH analysis (adjusted HR, 39.2; *p* = 0.011; p-value for trend = 0.013; Table 3). Regarding the composite adverse outcome, the Kaplan‒Meier analysis also showed a much higher event rate in the high MEHP group than in the other groups (log-rank test *p* = 0.001 and p-value for trend = 0.027; Fig. 2B). Moreover, multivariate-adjusted restricted cubic spline Cox PH regression also showed that the adjusted HR for the composite adverse outcome became significantly increased when the circulating MEHP concentration was higher than the cutoff value (Fig. 3). Furthermore, the high MEHP group had a significant 13-fold HR for the composite adverse outcome compared with the reference group in the multivariate Cox PH analysis (*p* = 0.001; p-value for trend = 0.008; Table 3).

**Table 3:** Association of mortality and adverse clinical outcomes with three groups regarding MEHP concentrations

	All-cause mortality				Composite adverse outcome^a^			
	Crude HR	p-value	Adjusted HR^b^	p-value	Crude HR	p-value	Adjusted HR^b^	p-value
Low MEHP	1.26 (0.11–13.9)	0.85	0.41 (0.02–8.76)	0.57	1.51 (0.36–6.33)	0.57	1.04 (0.20–5.28)	0.99
Modestly elevated MEHP	Reference	—	Reference	—	Reference	—	Reference	—
High MEHP	10.4 (1.90–56.6)	0.007	39.2 (2.34- 657)	0.011	6.45 (1.97–21.2)	0.002	13.0 (2.74–61.4)	0.001
p-value for trend		0.016		0.013		0.029		0.008

Abbreviations: HR, hazard ratio; MEHP, mono-2-ethylhexyl phthalate

^a^ The composite adverse outcome includes hospital admission due to cardiovascular disease, heart failure, stroke, infection, or cancer, and all-cause mortality

^b^ Adjusted for DEHP, IS, age, gender, diabetes, hypertension, cardiovascular disease, dialysis vintage, dialysis adequacy (Kt/V), and serum albumin

## Discussion

For the first time, we demonstrated the association of circulating MEHP with subsequent adverse outcomes in hemodialysis patients. In addition, circulating MEHP concentrations were positively and linearly correlated with DEHP concentrations. Moreover, serum MEHP concentration was an independent risk factor for all-cause mortality and composite adverse outcomes. We also found a cutoff value of MEHP for predicting mortality and composite adverse outcomes. Hemodialysis patients with circulating MEHP above the cutoff value had much higher risks for mortality and composite adverse outcomes.

Phthalates are easily released from cosmetics, perfumes, shampoos, paints, and other plastic products, including hemodialysis instruments. Phthalates are metabolized to monoesters by esterase and lipase mainly in the intestines, conjugated by uridine 5'-diphospho-glucuronosyltransferase to form the hydrophilic glucuronide conjugate, and finally excreted into the urine (Zhang et al. 2021). However, urinary excretion of phthalates is substantially diminished in patients undergoing chronic dialysis.

Phthalate exposure has been linked to several disorders involving the respiratory system (Yu and Wang 2022), the immune system and allergies (Bolling et al. 2020), the endocrine system, tumorigenesis, and the cardiovascular system. Phthalates could worsen pulmonary function and aggravate airway inflammation in asthmatic children (Kim et al. 2018). DEHP is one of the most widely used phthalates in commerce and MEHP is the most studied metabolite of it. Growing evidence has shown a variety of toxicities of MEHP to the human body. First, MEHP could negatively impact the endocrine, metabolism, and reproductive systems. Previous studies have shown that MEHP can cause endocrine disruption and metabolic disorders in animal and in vitro studies (Hao et al. 2012; Park et al. 2020). A recent study found that high concentrations of MEHP decrease DNA methylation in blastocysts and may negatively regulate gene expression and impact embryo development (Arcanjo et al. 2023). Second, MEHP could promote tumorigenesis and metastasis. MEHP promotes proliferation, migration, invasion, epithelial-mesenchymal transition, and metastasis in cancer cell lines and in nude mice (Leng et al. 2021; Yao et al. 2012). A recent study demonstrated that childhood exposure to phthalates was associated with higher risks of osteosarcoma and lymphoma before adulthood (Ahern et al. 2022). Third, MEHP may increase the susceptibility to infectious diseases. MEHP suppresses interleukin-23-mediated antiviral responses and may promote dengue virus infection (Lin et al. 2021). Moreover, growing evidence has shown that MEHP is linked to adverse cardiovascular outcomes such as hypertension, atherosclerosis, coronary artery disease, arrhythmia, and myocardial infarction (Mariana et al. 2023). Our results are in agreement with these previous studies and indicate that MEHP is a significant concern for health in chronic HD patients.

We reported that DEHP abolishes the beneficial effects of statins in patients on peritoneal dialysis and in endothelial cells (Guo et al. 2020). Tereshchenko et al. also proposed that phthalates such as DEHP could interact with abnormal electrophysiological substrates and increase the risk of sudden cardiac death (Tereshchenko and Posnack 2019). As the major active metabolite of DEHP, MEHP can induce apoptotic injury in endothelial cells through reactive oxygen species-mediated and mitochondria-dependent pathways (Ban et al. 2014). The toxic potency of MEHP could be 10 times higher than that of DEHP (Zhou et al. 2023). Recently, a large-scale population-based cohort study showed that MEHP exposure is linked to a significantly increased risk for cardiovascular mortality (Trasande et al. 2022). In addition, previous studies have shown that MEHP induces cytokine release (e.g., tumor necrosis factor-alpha) and inflammasome activation and may exaggerate inflammatory disorders (Bolling et al. 2012; Park et al. 2019). High levels of prenatal exposure to phthalates were reported to be associated with a decreased skeletal muscle index in children in a prospective cohort study (Lee et al. 2020). MEHP could alter mitochondrial function and homeostasis in skeletal muscle cells (Chen et al. 2020). This may aggravate sarcopenia, frailty, and fracture and cause disability in dialysis patients. Taken together, these underlying mechanisms of MEHP could contribute to the higher mortality rate and composite adverse events in hemodialysis patients.

We reported in a recent study that DEHP exposure higher than 68.7 ng/mL in serum abolished the protective effect of statins on cardiovascular disease in patients on peritoneal dialysis (Guo et al. 2020). To the best of our knowledge, no study has addressed the impact of MEHP on all-cause mortality or the composite endpoint of mortality and hospitalization due to cardiovascular disease, heart failure, stroke, and cancer in hemodialysis patients. We found that the impact of MEHP exposure on all-cause mortality and composite adverse outcomes had a trend in a dose-dependent manner. Additionally, our study revealed a cutoff value (41.8 ng/mL) of serum MEHP concentration for predicting these crucial outcomes in hemodialysis patients. We believe that MEHP exposure higher than the cutoff value might not be well tolerated by hemodialysis patients and would lead to subsequent adverse clinical outcomes. Therefore, it should be emphasized that developing guidelines or strategies to reduce phthalate exposure would be important not only for children and teenagers but also for dialysis patients.

This study also investigated the relationship between circulating phthalates and an important protein-bound uremic toxin – IS. Growing evidence shows that IS is an important risk factor for cardiovascular disease, heart failure, peripheral vascular disease, and mortality in hemodialysis patients (Cao et al. 2015; Hung et al. 2017; Lin et al. 2020). In the current study, circulating IS concentrations were not significantly correlated with concentrations of phthalates. Moreover, circulating IS was adjusted in the multivariate Cox PH regression. The adverse impact of MEHP exposure may be independent of the protein-bound uremic toxin in patients on hemodialysis.

There are limitations to this study. First, the study's sample size was small, and the event rate was low due to a short follow-up period (< 400 days). However, the association between circulating MEHP and adverse outcomes was obviously observed in this study. Second, there may be unmeasurable confounders in the current study, although we tried our best to adjust many important risk factors for adverse clinical outcomes in hemodialysis patients. In addition, we need validation cohorts for the cutoff value of MEHP in the future. Third, we did not repeat measurements of circulating phthalates during the follow-up period. The trajectories of circulating phthalates (whether accumulation or a surge) may provide additional prediction values for predicting clinical outcomes. Finally, our study only recruited Taiwanese individuals. Whether our results can be generalized to other ethnicities needs further studies for validation.

## Conclusions

Circulating MEHP is an independent risk factor for mortality and other adverse outcomes in hemodialysis patients. Blood MEHP concentrations greater than 41.8 ng/mL signal poor prognosis in this population. Because of limitations (e.g., small sample size, relatively short follow-up, and exclusively Taiwanese population) in the current study, further research is needed to confirm and validate our results in the future. Furthermore, future research directions would be the underlying mechanisms of MEHP contributing to adverse outcomes and exploring the vulnerable sub-populations to enhance the care for dialysis patients.

### Supplementary Information

Below is the link to the electronic supplementary material.

Supplementary file1 (DOCX 484 KB)

## Data Availability

The datasets used or analyzed during the current study are available from the corresponding author on reasonable request.

## Abbreviations

CI: Confidence interval
CKD: Chronic kidney disease
DEHP: Di-2-ethylhexyl phthalate
ESRD: End-stage renal disease
HR: Hazard ratio
IQR: Interquartile range
IS: Indoxyl sulfate
MEHP: Mono-2-ethylhexyl phthalate
PH: Proportional hazard
ROC: Receiver operating characteristic
SD: Standard deviation
