# Supplementary material for: Association of mono-2-ethylhexyl phthalate with adverse outcomes in chronic hemodialysis patients
Source: Environ Sci Pollut Res Int. 2023 Nov 8;30(57):120366–74. doi: 10.1007/s11356-023-30814-z (PMC10697867; doi:10.1007/s11356-023-30814-z)
Supplement: Supplementary file 1 — Supplementary file1 (DOCX 484 KB) [file 11356_2023_30814_MOESM1_ESM.docx]

**Supplementary Materials**

**Figure S1.** Flowchart of patient selection.

**Figure S2.** Evaluation of the risk model for predicting mortality by using Harrell’s C-index.

**Figure S3.** Evaluation of the risk model for predicting composite adverse outcomes by using Harrell’s C-index.

**Figure S4.** Time-dependent receiver operating characteristic curve for determining the cutoff value in terms of censored survival data.

**Figure S1.** Flowchart of patient selection.

Abbreviations: DEHP, di-2-ethylhexyl phthalate; HD, hemodialysis; IS, indoxyl sulfate; MEHP, mono-2-ethylhexyl phthalate.

**Figure S2.** Evaluation of the risk model for predicting mortality by using Harrell’s C-index.


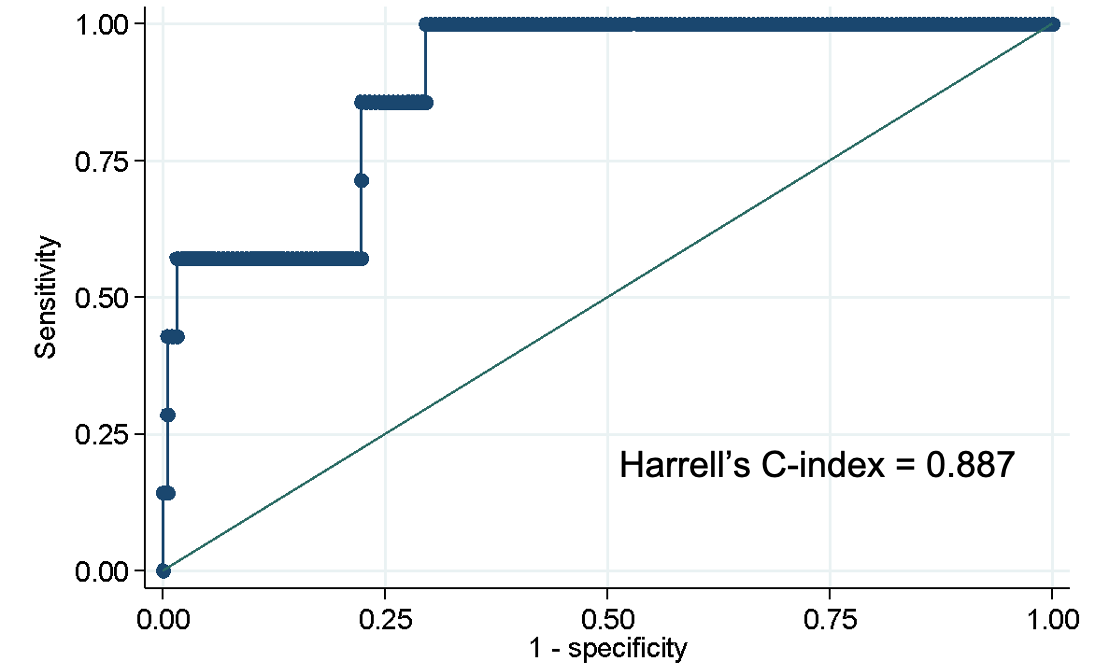


The risk model includes DEHP, MEHP, IS, age, gender, diabetes mellitus, hypertension, cardiovascular disease, dialysis vintage, Kt/V, and albumin.

Abbreviations: DEHP, di-2-ethylhexyl phthalate; IS, indoxyl sulfate; MEHP, mono-2-ethylhexyl phthalate.

**Figure S3.** Evaluation of the risk model for predicting composite adverse outcomes by using Harrell’s C-index.


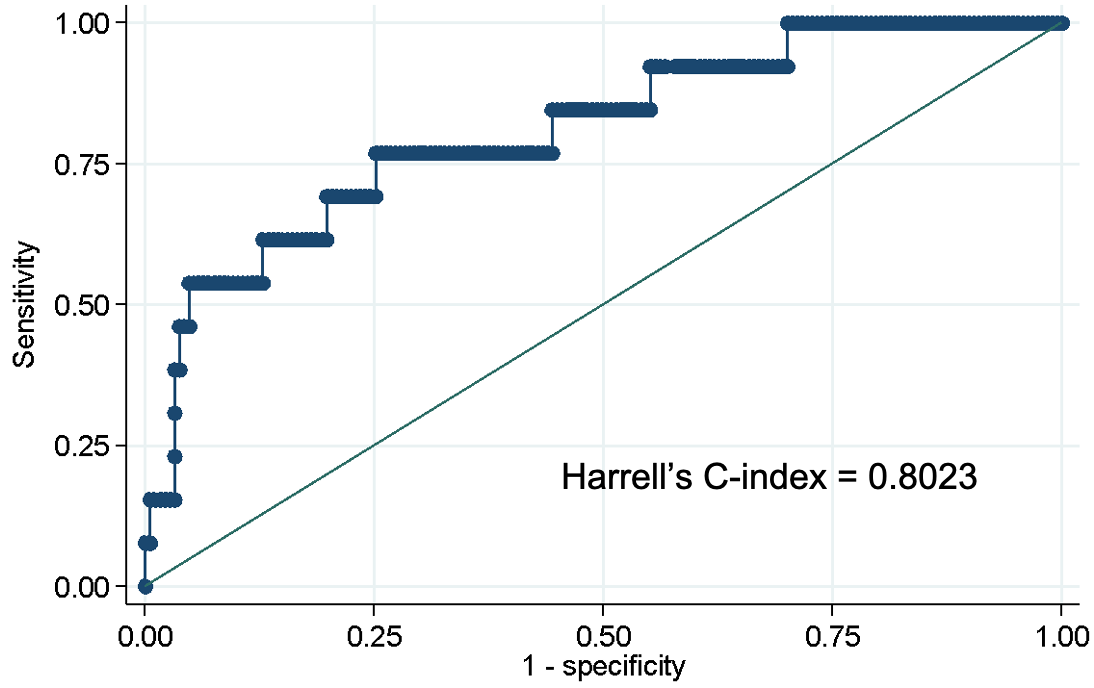


The risk model includes DEHP, MEHP, IS, age, gender, diabetes mellitus, hypertension, cardiovascular disease, dialysis vintage, Kt/V, and albumin.

Abbreviations: DEHP, di-2-ethylhexyl phthalate; IS, indoxyl sulfate; MEHP, mono-2-ethylhexyl phthalate.

**Figure S4.** Time-dependent receiver operating characteristic curve for determining the cutoff value in terms of censored survival data.


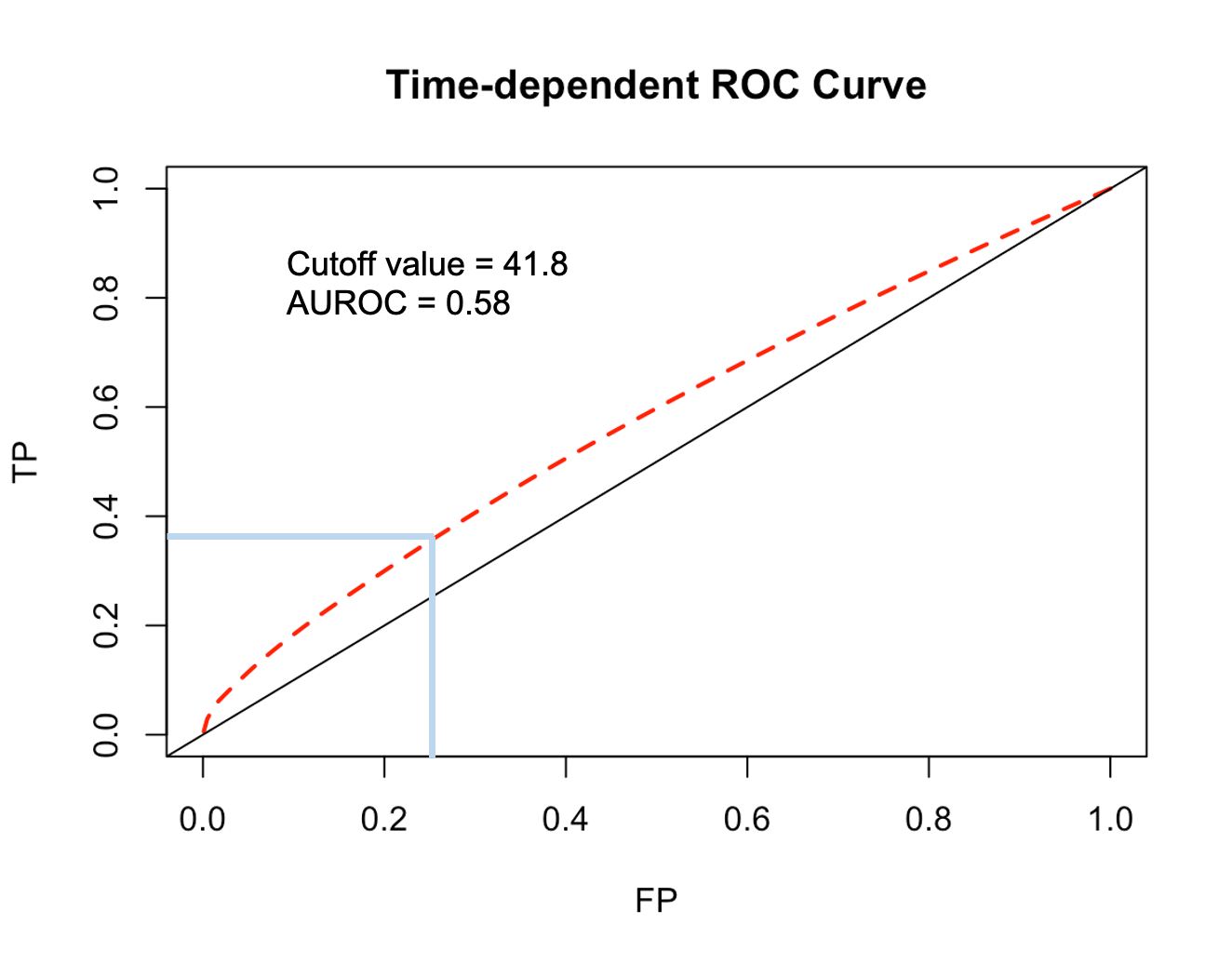


The cutoff value of MEHP was determined by calculating the maximum TP minus FP values under the time-dependent ROC curve.

Abbreviations: AUROC, area under ROC curve; FP, false positive rate; MEHP, mono-2-ethylhexyl phthalate; ROC, receiver operating characteristic; TP, true positive rate.
